# Effectiveness of acupuncture for treatment of diabetic peripheral neuropathy

**DOI:** 10.1097/MD.0000000000017282

**Published:** 2019-09-27

**Authors:** You-jie Zhang, Fan-rong Liu

**Affiliations:** a Department of Integrated Chinese and Western Medicine, Shangluo Central Hospital; b Department of Gastroenterology, Shangluo Central Hospital, Shangluo, China.

**Keywords:** acupuncture, diabetic peripheral neuropathy, effectiveness, randomized controlled trial, safety

## Abstract

**Background:**

This study will assess the effectiveness and safety of acupuncture for the treatment of patients with diabetic peripheral neuropathy (DPN).

**Methods:**

We will comprehensively search electronic databases of MEDLINE, EMBASE, Cochrane Library, Web of Science, Chinese Biomedical Literature Database, Chinese Scientific Journal Database, and China National Knowledge Infrastructure from their inception to July 1, 2019. We will also search grey literature to avoid missing any potential studies. Randomized controlled trials related to acupuncture for the treatment of DPN will be included. All record literatures are searched without language limitation. Two researchers will independently carry out research selection, data extraction, and research quality evaluation. We will perform RevMan 5.3 software for statistical analysis.

**Results:**

Primary outcomes consist of severity of neuropathy and pain intensity. Secondary outcomes include diabetes mellitus duration, body mass index, HbA1c level, blood glucose levels, and adverse events.

**Conclusion:**

The findings of this study will summarize recent evidence for the effectiveness and safety of acupuncture for the treatment of patients with DPN.

**Ethics and dissemination:**

We will not analyze individual data, thus no ethic approval is needed. The results of this study are expected to be published at a peer-reviewed journal.

**PROSPERO registration number:**

PROSPERO CRD42019139635.

## 1 Introduction

Diabetic peripheral neuropathy (DPN) is one of the most common complications in patients with diabetes mellitus (DM).^[1–3]^ Such condition often affects sensory, autonomic, and motor nerve functions.^[4,5]^ It has been estimated that it affects about 30% to 50% of all DM patients, and about 16% to 26% of people with DM suffer from painful peripheral neuropathy.^[6–8]^ It is also reported that about 7% of those patients with foot ulceration may need amputation within 10 years.^[9]^ If it cannot be treated fairly well, it can result in foot ulceration and amputation,^[10]^ and can greatly affect quality of life in patients with such condition.^[11]^

Presently, there is no specific curative pharmacologic management for patients with DPN, and no ideal clinical efficacy has been achieved. Those patients mainly accept comprehensive treatments, such as physical training, vitamin B supplementation, Chinese herbal medicine, moxibustion, and acupuncture.^[12–19]^ Of these, acupuncture has been reported to treat DPN effectively.^[20–25]^ However, no systematic review has been conducted to assess its effectiveness and safety for patients with DPN. Therefore, in this study, we will investigate the effectiveness and safety of acupuncture for patients with DPN.

## 2 Methods

### 2.1 Eligibility criteria

#### 2.1.1 Study types

Randomized controlled trials (RCTs) on assessing the effectiveness and safety of acupuncture for the treatment of DPN will be considered for inclusion. We will exclude studies of non-clinical studies, and non-RCTs.

#### 2.1.2 Participant types

All patients with diagnosed DPN will be considered for inclusion, regardless the race, gender, age, and educational status.

#### 2.1.3 Intervention types

In the experimental group, patients can receive acupuncture treatment alone.

In the control group, patients can receive any treatments, except any forms of acupuncture.

#### 2.1.4 Outcome types

Primary outcomes consist of severity of neuropathy, as measured by any related scales, such as Michigan Diabetic Neuropathy Score; and pain intensity, as measured by numerical rating scale, and any associated scales.

Secondary outcomes include DM duration, body mass index, HbA1c level, blood glucose levels, and adverse events.

### 2.2 Data sources and search methods

This study will search electronic databases of MEDLINE, EMBASE, Cochrane Library, Web of Science, Chinese Biomedical Literature Database, Chinese Scientific Journal Database, and China National Knowledge Infrastructure from the date of creation to July 1, 2019. We will also search grey literature, such as conference proceedings, and reference lists of included studies. The example search strategy for Cochrane Library is showed in Table 1. This search strategy will be modified and applied to other electronic databases.

**Table 1:**
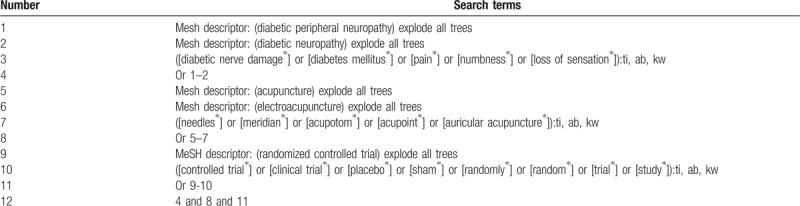
Search strategy applied in Cochrane Library database.

### 2.3 Data collection

#### 2.3.1 Study selection

**Figure 1:**
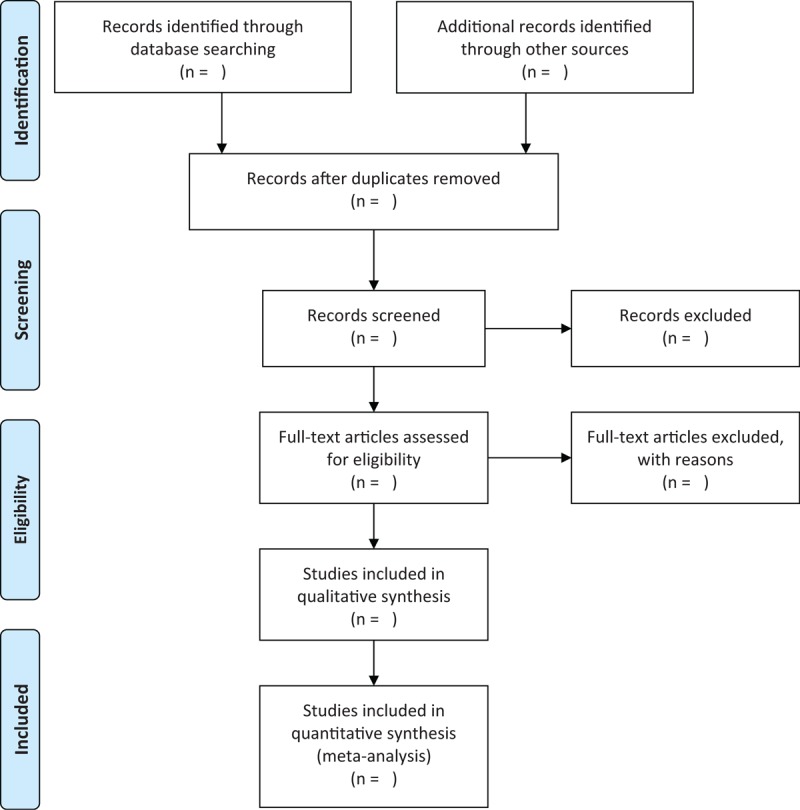
Flowchart of study selection.

After all search work complete, the NoteExpress 3.2.0 software will be utilized for study selection, and all repetitive studies will be removed. The whole process of study selection will be performed independently by two reviewers, and will determine the final inclusion of these records. Any disagreements will be solved by a third reviewer. At the first stage, all titles and abstracts will be identified to check if they meet the selection criteria. At the second stage, we will assess the full text of remaining studies and determine whether it is eligible for inclusion criteria. The search flow chart will be shown in Figure 1.

#### 2.3.2 Data extraction

Data extraction and analysis will be conducted by two independent researchers. If the differences and opinions are inconsistent, a third researcher will help to solve these differences and opinions by discussion. We will extract literature data of title, first author, region, year of publication, study characteristics, patient characteristics, sample size, study design, study methods, intervention details, outcome measurements, safety, conflicts of interest, and other information. If the reported data is insufficient or missing, we will contact primary authors of original clinical RCTs.

### 2.4 Assessment of risk of bias

Two independently researchers will assess methodological quality using Cochrane Risk of Bias Tool. An experienced third researcher will help to settle down any divisions between two authors. The contents include 7 items, and each one is divided into 3 levels: low risk, high risk, and uncertain risk.

### 2.5 Data synthesis and analysis

Data synthesis will be carried out using ReMan 5.3 software. The results will be expressed as follows: continuous data for mean difference or standardized mean difference with 95% confidence intervals, and dichotomous data for risk ratio with 95% confidence intervals.

*I*^2^ test will be used for heterogeneity check, and it is interpreted as below: *I*^2^ ≤ 50 indicating satisfied heterogeneity and a fixed-effects model will be utilized; *I*^2^ > 50% indicating high heterogeneity and a random-effects model will be applied. If the heterogeneity is satisfied, meta-analysis will be conducted. If the heterogeneity is high, we will explore the possible reasons from subgroup analysis performance. If heterogeneity is still high after subgroup analysis, the data cannot be synthesized, and we will only summarize descriptive analysis.

### 2.6 Additional analysis

We will perform subgroup analysis according to the different study characteristics, study treatments, and outcomes. Additionally, we will also conduct sensitivity analysis to identify the robustness of outcome results by removing low quality studies.

### 2.7 Reporting bias

We will also conduct Funnel plot^[26]^ and Egger's regression test^[27]^ to explore reporting bias when more than 10 RCTs entered.

## 3 Discussion

DPN is a very server disorder, and often causes high morbidity, and greatly affects quality of life in patients with such condition. Although several studies have reported acupuncture can be used to treat DPN, no study assesses the efficacy and safety of acupuncture for the treatment of patients with DPN. Therefore, a standardized and detailed protocol is great importance to provide significant evidence-based findings of acupuncture for DPN. This study intends to evaluate its efficacy and safety for DPN. The results of this study may provide rigorous summary evidence of acupuncture for the treatment of patients with DPN across all published RCTs.

## Acknowledgments

This study is supported by Science and Technology Research Project of Xianyang City (2016k02–101). The funder had no role in this study.

## Author contributions

**Conceptualization:** You-jie Zhang, Fan-rong Liu.

**Data curation:** You-jie Zhang, Fan-rong Liu.

**Formal analysis:** You-jie Zhang.

**Funding acquisition:** Fan-rong Liu.

**Investigation:** Fan-rong Liu.

**Methodology:** You-jie Zhang.

**Project administration:** Fan-rong Liu.

**Resources:** You-jie Zhang.

**Software:** You-jie Zhang.

**Supervision:** Fan-rong Liu.

**Validation:** You-jie Zhang, Fan-rong Liu.

**Visualization:** You-jie Zhang.

**Writing – original draft:** You-jie Zhang, Fan-rong Liu.

**Writing – review & editing:** You-jie Zhang, Fan-rong Liu.
